# Is Branched-Chain Amino Acids Supplementation an Efficient Nutritional Strategy to Alleviate Skeletal Muscle Damage? A Systematic Review

**DOI:** 10.3390/nu9101047

**Published:** 2017-09-21

**Authors:** Alexandre Fouré, David Bendahan

**Affiliations:** Aix Marseille University, CNRS, Centre de Résonance Magnétique Biologique et Médicale (CRMBM), UMR 7339, Faculté de Médecine la Timone, 27 Boulevard Jean Moulin, 13385 Marseille, France; david.bendahan@univ-amu.fr

**Keywords:** branched-chain amino acids (BCAAs), exercise-induced muscle damage, skeletal muscle, nutritional strategy

## Abstract

Amino acids and more precisely, branched-chain amino acids (BCAAs), are usually consumed as nutritional supplements by many athletes and people involved in regular and moderate physical activities regardless of their practice level. BCAAs have been initially shown to increase muscle mass and have also been implicated in the limitation of structural and metabolic alterations associated with exercise damage. This systematic review provides a comprehensive analysis of the literature regarding the beneficial effects of BCAAs supplementation within the context of exercise-induced muscle damage or muscle injury. The potential benefit of a BCAAs supplementation was also analyzed according to the supplementation strategy—amount of BCAAs, frequency and duration of the supplementation—and the extent of muscle damage. The review protocol was registered prospectively with Prospective Register for Systematic Reviews (registration number CRD42017073006) and followed Preferred Reporting Items for Systematic reviews and Meta-Analyses guidelines. Literature search was performed from the date of commencement until August 2017 using four online databases (Medline, Cochrane library, Web of science and ScienceDirect). Original research articles: (i) written in English; (ii) describing experiments performed in Humans who received at least one oral BCAAs supplementation composed of leucine, isoleucine and valine mixture only as a nutritional strategy and (iii) reporting a follow-up of at least one day after exercise-induced muscle damage, were included in the systematic review analysis. Quality assessment was undertaken independently using the Quality Criteria Checklist for Primary Research. Changes in indirect markers of muscle damage were considered as primary outcome measures. Secondary outcome measures were the extent of change in indirect markers of muscle damage. In total, 11 studies were included in the analysis. A high heterogeneity was found regarding the different outcomes of these studies. The risk of bias was moderate considering the quality ratings were positive for six and neutral for three. Although a small number of studies were included, BCAAs supplementation can be efficacious on outcomes of exercise-induced muscle damage, as long as the extent of muscle damage was low-to-moderate, the supplementation strategy combined a high daily BCAAs intake (>200 mg kg^−1^ day^−1^) for a long period of time (>10 days); it was especially effective if taken prior to the damaging exercise.

## 1. Introduction

In a recent report entitled “Protein Ingredients Market by Source (Animal and Plant), Application (Food & Beverage, Animal Feed, Cosmetics & Personal Care and Pharmaceuticals), and Region—Forecast to 2022”, the market for protein ingredients was projected to reach 58.49 billion dollars by 2022 (i.e., compound annual growth rate of 6.0% from 2017). Indeed, Olympic as well as college athletes and many people exercising in gyms regularly use supplements with amino acids representing 10–20% of these nutritional strategies [1,2,3,4]. Branched chain amino acids (BCAAs)—i.e., leucine, isoleucine and valine—account for almost 50% of the essential amino acids in food and 35% of the total content of essential amino acids in muscle proteins [5,6].

BCAAs are important precursors of tricarboxylic acid (TCA) cycle intermediates via acetyl-CoA and Succinyl-CoA [7] and can be involved in energy production through the modulation of exercise-induced serum BCAAs oxidation [8]. In addition to their involvement as constitutive elements of the structural and contractile proteins synthesis [9], BCAAs are also considered signaling molecules [10]. Indeed, BCAAs and especially leucine have been reported to activate the mammalian target of rapamycin signaling pathway [11,12], thereby promoting muscle-protein synthesis [13,14,15]. It has also been suggested that they could enhance mitochondrial biogenesis and reactive oxygen species scavenging [7,16] leading to potential benefits in skeletal muscle energy metabolism [17,18,19].

Taking into account all the physiological mechanisms linked to BCAAs intake and that BCAAs are mainly metabolized in skeletal muscle [5], whereas other essential amino acids are catabolized in liver [20]; BCAAs supplementation has been considered as a potential nutritional strategy to avoid or at least alleviate exercise-induced muscle damage or its consequences. Exercise-induced muscle damage (EIMD) has been primarily associated with mechanical strain [21,22] and the subsequent inflammation processes [23]. On that basis, it has been considered that diminished muscle-protein breakdown during exercise [24] and the scavenging of reactive oxygen species [7] could alleviate structural and metabolic alterations observed after EIMD [25,26]. In addition, the anabolic effect associated with BCAAs consumption and especially leucine [9,27] has been considered a potential promoter of the repair process of altered muscle tissues in part composed of proteins. However, so far, no imaging study has ever reported direct evidence supporting these assumptions (e.g., [28]). Therefore, the occurrence of muscle damage in all studies considered in this systematic review was assessed on the basis of EIMD outcomes, including muscle function alteration (i.e., force loss), increased blood markers of muscle damage (i.e., creatine kinase (CK), lactate dehydrogenase (LDH) and myoglobin) and delayed onset muscle soreness.

The aim of this systematic review was to objectively describe the effects of BCAAs supplementation on indirect markers of muscle damage considering studies reporting clinical trials (cross-over design and randomized clinical trials with a control group) involving healthy subjects supplemented with BCAAs only. Potential confounding factors were determined relating to the extent of muscle damage (i.e., low, moderate and high) and the supplementation strategy (i.e., duration, frequency and amount).

This systematic review provides a comprehensive analysis of the literature regarding the assumption that BCAAs supplementation can alleviate alterations of skeletal muscle function acting on exercise-induced muscle damage or muscle injury. Recommendations on the most efficient nutritional strategy to minimize consequences of damage induced by exercise on muscle function are highlighted.

## 2. Methods

The pre-defined review protocol was registered prospectively with Prospective Register for Systematic Reviews (PROSPERO—registration number: CRD42017073006). This systematic review was completed in accordance with the recommendations of the Preferred Reporting Items for Systematic reviews and Meta-Analyses (PRISMA) guidelines [29].

### 2.1. Eligibility Criteria

Criteria for study inclusion were chosen using the Population-Intervention-Comparator-Outcomes-Study design (PICOS) format [29]. Articles and studies were included if they met all the following criteria: (1) experiments performed in humans; (2) healthy subjects received at least one oral BCAAs supplementation as a nutritional strategy in the context of skeletal muscle damage (i.e., decrease in muscle performance and/or increase in plasma/serum intracellular component concentration); (3) supplementation only composed of leucine, isoleucine and valine; (4) follow up performed at least one day after exercise-induced muscle damage or muscle injury; (5) original research articles; and (6) written in English. Studies were excluded if the experimental group(s) undertook any other practice that could be perceived as a strategy to alleviate muscle damage (e.g., massage, cryotherapy). Outcome measures were changes in indirect markers of muscle damage, i.e., muscle function performance (isometric force, jump height) and plasma/serum concentration of intracellular components (creatine kinase, lactate dehydrogenase, myoglobin). Clinical trials using a control group or a cross over design were included in the systematic review.

### 2.2. Search Strategy

The computerized literature search was performed from date of commencement until August 2017 using four online databases: Medline (PubMed), Cochrane library, Web of Science and ScienceDirect; a supplementary Google Scholar search was also undertaken. The key words used to find relevant papers were: (“muscle damage” OR “muscle injury” OR “exercise-induced muscle damage” OR EIMD) AND (“nutritional strategy” OR “branched-chain amino acid” OR BCAA OR supplementation). The reference sections of all identified articles were also examined.

### 2.3. Data Extraction and Quality Assessment

Data related to participants (sex, sample size, age), experimental design (randomization, blinding, wash-out period in case of cross-over design, dietary control), exercise (intensity, volume and type of exercise), outcome measures (muscle performance and blood analyses) and supplementation strategy (duration, frequency, daily intake of BCAAs, relative concentration of leucine/isoleucine/valine) were extracted. The quality of selected studies (i.e., corresponding to all eligibility criteria) were rated using the Quality Criteria Checklist for Primary Research [30] to limit the risk of bias. Significant and non-significant results were also exhaustively reported to objectively assess effects of each supplementation strategy (combining duration, frequency and amount of daily BCAAs intake) on the extent of muscle damage.

## 3. Analysis

A greater emphasis was placed on findings from studies achieving high-quality ratings. Significant effects described in the included studies were extracted to quantify outcomes associated with the damaging exercise and the BCAAs supplementation. Rating criteria were created according to the supplementation strategy and the extent of muscle damage (Table 1). Due to the heterogeneity of the study designs, interventions and outcomes, a meta-analysis was not undertaken.

## 4. Results

**Figure 1:**
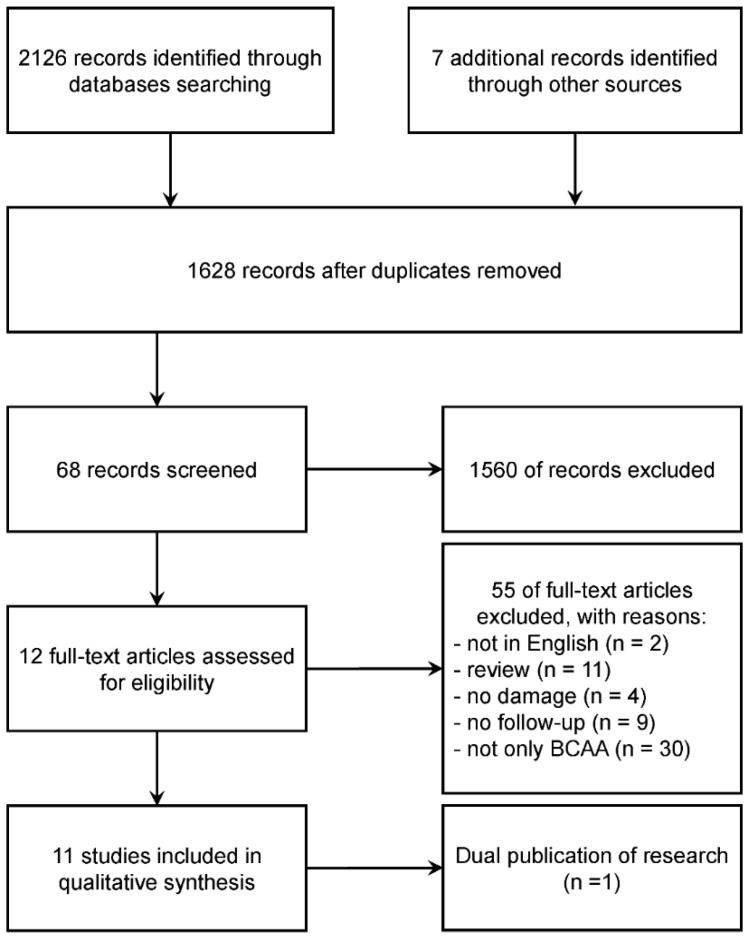
Study selection and flow diagram of articles included in the systematic review.

We initially identified two thousand one hundred and thirty-three papers from databases and internet searches and included 11 studies in the present systematic review according to the 4-phase flow diagram described in Figure 1.

The included studies were trials with numbers of subjects ranging from 9 to 30 and were conducted in the last 20 years (Table 2). We identified large heterogeneity regarding supplementation strategies and damaging exercise modalities leading to a large variability in the damage extents.

### 4.1. Study Quality

The majority of studies included in the systematic review were rated as positive (55%). Neutral (27%) and negative (18%) qualities were reported for the other studies (Table 3) for multiple reasons including a cross-over design without a control group [33,34,38,40], the lack of efficient randomization and blinding [31,33,34,36,37,40] and the absence of statements on funding and sponsorship [31,34,38,40]. In addition, short follow-up of exercise-induced muscle damage outcomes (<2 days) was also observed in studies rated as negative [33,38].

Moreover, the supplementation strategy and the extent of EIMD were considered as cofounding parameters to objectively assess the effects of BCAAs on muscle damage outcomes.

### 4.2. Supplementation Strategy and Muscle Damage Extent

Duration, frequency and daily amount of BCAAs was rated (Table 4). More than half of the studies reported a short (≤3 days) duration of supplementation whereas the frequency and the daily amount of BCAAs intakes (from low to high) was similarly distributed among the included studies.

The damage extent was generally low in the included studies. It is noteworthy that a few studies reported discordant changes in muscle performance and CK/LDH measurements [36,40] as a result of EIMD (i.e., large decrease in force and no/small change in plasma CK).

### 4.3. Outcomes

The positive effects of BCAAs supplementation on EIMD outcomes are reported in Table 5. The number of studies demonstrating a positive effect was equivalent to the number of studies which showed no effect. It should be noted that a positive effect was clearly reported by the lower quality studies whereas positive quality studies described no significant effect except for the results from Howatson et al. [35].

Considering the studies with positive and neutral quality rating, the benefits of BCAAs supplementation was mostly observed when the supplementation strategy included a high amount of BCAAs intake (>200 mg kg^−1^ day^−1^) in a context of low-to-moderate muscle damage extent [31,33,35,38]. In addition, a high frequency of BCAAs intake (2 or more daily intakes) and a long duration of supplementation (>10 days) and even more on several days before the damaging exercise (at least 7 days prior to the damaging exercise in the two studies showing a positive effect of BCAAs supplementation) appears to alleviate outcomes of EIMD [31,35].

## 5. Discussion

In the last few years, nutritional strategy has been considered crucial for the optimization of muscle performance. More particularly, BCAAs supplementation has been used in the field of sports with the aims of limiting the outcomes (e.g., force loss) of EIMD. Throughout this systematic review, we identified that BCAAs can alleviate outcomes of EIMD for specific conditions regarding the extent of muscle damage and the supplementation strategy. Potential benefits of BCAAs supplementation can actually be obtained for low-to-moderate extent of muscle damage and considering a supplementation strategy that includes high daily BCAAs intake over a long period of time (i.e., several days) and especially before the damaging exercise period.

Previous systematic reviews have reported positive chronic effects of protein supplementation on muscle mass, strength and power [42]. The corresponding physiological mechanisms—i.e., a decreased muscle-protein breakdown [24] and a reactive oxygen species scavenging [7]—could also lead to improved muscle performance and be beneficial in alleviating muscle damage. However, the efficiency of the latter mechanisms could require time thereby explaining the potential need for long-lasting supplementation prior to EIMD in order to obtain potential benefits.

### 5.1. Extent of Exercise-Induced Muscle Damage

A first confounding factor for the assessment of BCAAs supplementation was the extent of muscle tissue alteration reported in the included studies. These alterations and the corresponding extent were estimated and rated from changes in indirect markers of muscle damage including decreased muscle performance and increased amount of blood markers (i.e., plasma/serum CK, LDH and/or myoglobin). As reported previously, the maximal voluntary contraction (MVC) loss is currently considered as the most reliable indicator of muscle injury [43] as compared to CK measurements. Indeed, a high inter-subject variability was found in plasma/serum CK level changes resulting from EIMD [25,36]. In “high-responder” subjects—i.e., those with the higher CK levels—the increased plasma/serum CK levels were then uncorrelated to the extent of muscle damage [21,44]. However, despite the variability of this outcome, it remains moderately correlated to muscle alterations assessed with MRI [45]. Therefore, we considered in the present systematic review that the combination of changes in force and blood markers both led to an objective assessment of muscle damage extent.

Muscle soreness is a subjective outcome of EIMD [46,47] given that it is strongly related to the subject’s previous experience with muscle damage. In the papers selected in the present systematic review, soreness was quantified as an outcome of EIMD in order to detect onset of muscle damage [21] but was not taken into account to estimate the extent of the corresponding alterations [48]. A methodological limitation can also be addressed regarding the assessment of muscle damage on the basis of MVC measurements. Indeed, MVC could not be considered a reliable marker to assess muscle damage extent taking into account the peripheral and central nervous alterations demonstrated in the first days after the damaging exercise [45,49,50,51,52]. Ideally, imaging methods such as electron microscopy, MRI or ultrasound elastography could be used to visualize the extent and assess the severity of muscle damage [28,53,54]. Most of the studies included in this review did not use these imaging methods in order to assess muscle damage and it has been sometimes difficult to assess muscle damage extent from changes in blood markers and muscle performance.

Putting aside these methodological considerations, positive effects of BCAAs supplementation have been mainly reported for low-to-moderate muscle damage induced by exercise. For larger muscle alterations, which have been reported in two studies [32,35], no significant effect of BCAAs supplementation has been disclosed. In these latter studies, the physiological benefits commonly linked to BCAAs supplementation—i.e., promotion of muscle-protein synthesis, reduction of protein oxidation, mitochondrial biogenesis and scavenging of reactive oxygen species—could not overcome the large alterations of muscle structural organization and/or muscle metabolism previously described [25,26,28,55,56]. However, muscle energetics impairment [57] and structural alterations [58,59,60] associated with low-to-moderate extent of muscle damage could be alleviated by a specific BCAAs supplementation strategy.

### 5.2. The Supplementation Strategy

In the present review, we considered three criteria (frequency, amount, duration) in order to more accurately assess the BCAAs supplementation strategies reported in the selected studies. A high frequency of BCAAs intake (i.e., two or more daily intakes per day) was used over the whole set of studies demonstrating benefits regarding EIMD. In addition, the daily amount of BCAAs and the supplementation duration seem to be important factors. Positive effects of BCAAs supplementation were mainly obtained for a high daily amount of BCAAs intake (>200 mg kg^−1^ day^−1^) over a long period of time [31,35]. However, the combination of these three criteria (i.e., frequency, amount and duration) appears critical to potentially trigger beneficial effects from BCAAs supplementation. For instance, considering low-to-moderate EIMD, a low frequency (i.e., less than 2 intakes per day) and a low daily amount of BCAAs intake (i.e., less than 200 mg kg^−1^ day^−1^) during a moderate supplementation duration (i.e., between 4 and 10 days) was not enough to produce benefits on EIMD indirect markers [37]. Moreover, no significant positive effects on outcomes has been found when supplementation combined a high frequency and a high daily amount over a short period of time [36,41]. A long BCAAs supplementation period (>10 days) appears necessary if one is expecting beneficial effects. Previous studies investigating the damaging effect of marathon on skeletal muscle used this rationale to assess the potential beneficial effects of BCAAs supplementation [61]. The authors reported no effect but did not investigate the delayed outcomes of EIMD and the study was thus excluded from the present systematic review. The inclusion/exclusion criteria we chose were relatively restrictive. In that way, several studies of interest were excluded to avoid additional confounding effects mainly associated with the composition of the supplementation mixture. A recent systematic review reported the global effects of protein supplementation [62].

Chronic BCAAs supplementation has been shown to produce positive effects in both animals and humans [7,16]. Increased skeletal muscle mitochondrial biogenesis and prevention of oxidative damage were described as potential mechanisms contributing to the increased lifespan in animals [16]. Therefore, BCAAs supplementation taken prior (days/weeks) to damaging exercise could prevent skeletal muscle tissues alterations through the enhanced mitochondrial biogenesis and reactive oxygen species scavenging [7]. This would occur via an upregulation of peroxisome proliferator-activated receptor-γ coactivator 1α expression [7,16].

## 6. Conclusions

In summary, the efficacy of a nutritional strategy based on BCAAs supplementation and aimed at reducing/preventing muscle damage resulting from high-intensity exercise seems to be poor. Among the studies selected in the present review, only one rated as positive regarding the quality of reported beneficial effects [35]. However, these beneficial effects should be considered with caution given the small sample size (*n* = 6) of both the control and supplemented groups. Overall, this systematic review suggests that a BCAAs supplementation strategy with daily intake larger than 200 mg kg^−1^ day^−1^, duration longer than 10 days starting at least 7 days before the damaging exercise would be effective to limit muscle damage resulting from exercise. On that basis, one can expect a peak force loss of less than 15% and/or an increase in plasma/serum CK peaking at one day following the damaging exercise. However, further placebo-controlled randomized clinical trials would be needed to support the beneficial effects of this strategy. In addition, it might be of interest to assay the effects of other nutritional strategies for which BCAAs could be combined with taurine [39] or other essential amino acids [63] and for which potentiator effects of BCAAs have been suggested.

As a take home message, there is no direct evidence of positive effects of BCAAs on muscle damage. However, in specific conditions, BCAAs supplementation seems to diminish the outcomes of EIMD. It would be of interest to further support these findings on the basis of imaging investigations.

## Figures and Tables

**Table 1:** Rating criteria concerning the supplementation strategy (i.e., duration, frequency and amount of daily BCAAs intake) and the extent of muscle damage assessed from changes in indirect markers of muscular alterations in the control group.

	Category	Rating	Criteria
**Supplementation Strategy**	**Duration**	Short	The supplementation was performed on 3 days or less
		Moderate	The supplementation was performed between 4 and 10 days
		Long	The supplementation was performed for more than 10 days
	**Frequency**	Low	Less than 2 intakes per day during the supplementation period
		High	2 or more intakes per day during the supplementation period
	**Amount**	Low	Less than 200 mg kg^−1^ day^−1^ of BCAAs intake
		High	200 mg kg^−1^ day^−1^ or more of BCAAs intake
**Extent of Muscle Damage**		Low	Low peak decrease in force (≤10% of baseline) and significant peak change in CK/LDH/myoglobin at D1 (with no significant difference in the following days)
		Moderate	Moderate peak decrease in force (≥10% and ≤15% of baseline) and significant peak change in CK/LDH/myoglobin at D1 (with significant difference in the following days)
		High	High peak decrease in force (>15% of baseline) and significant peak change in CK/LDH/myoglobin after D2

**Table 2:** Studies included in the systematic review.

Study	Population	Study Design	Damaging Exercise	Supplementation Strategy	Outcomes		
					Soreness	Blood Analysis (Myoglobin/LDH/CK)	Muscle Performance
Coombes & McNaughton (2000) [31]	16 healthy males age: 21 ± 1 years V̇O_2max_: 52 ± 4 mL min^−1^ kg^−1^ training status: regular physical activity	CG (*n* = 8) Dietary control	Cycling ergometer exercise at 70% V̇O_2max_ for 120 min	14 days of supplementation (7 days before and 6 days after exercise). 2 × 6 g every day + 20 g before and after the exercise Amount of BCAA: 208 g (14 days) LEU/ISO/VAL (1:1:1) Placebo: no supplementation	-	CG > SG at H4, D1, D3 and D5 for CK and LDH	-
Fouré et al. (2016) [32]	26 healthy males age: 22 ± 2 years training status: recreationally active	RCT, DB CG (*n* = 13) Dietary control	Neuromuscular Electrostimulation—40 isometric knee extensions	5 days of supplementation (2 supp before, 1 supp after exercise and 1 supp every day for 4 days) Amount of BCAA: 48.3 g (5 days) LEU/ISO/VAL (2:1:1) Placebo: microcrystalline cellulose	CG = SG	SG > CG at D4 for CK	CG = SG for the MVC
Gee & Deniel (2016) [33]	11 healthy males age: 25 ± 6 years training status: resistance-trained	RCT, SB Cross-over (washout: 7 days)	Strength exercises (back squat, press exercises, deadlift and barbell row)	Single day supplementation (1 supp before and 1 supp after exercise) Amount of BCAA: 20 g (1 day) LEU/ISO/VAL (2:1:1) Placebo: apple and blackcurrant juice	CG = SG	-	SG > CG at D1 for the CMJ and the SSPT
Greer et al. (2007) [34]	9 healthy males age: 22 ± 3 years V̇O_2max_: 36 ± 2 mL min^−1^ kg^−1^ training status: untrained	Cross-over (washout: 8 days) Dietary control	Cycling ergometer exercise at 55% V̇O_2max_ for 90 min	Single day supplementation (1 supp before and 1 supp at 60 min during the exercise) Amount of BCAA: 5 g (1 day) LEU/ISO/VAL (2.5:1:1.5) Placebo: water, lemon flavor, salts and artificial sweeteners.	CG > SG at D1	CG > SG at H4, D1 and D2 for CK CG > SG at H4 for LDH	SG > CG at D2 for leg flexion torque (180°/s)
Howatson et al. (2012) [35]	12 healthy males age: 23 ± 2 years training status: trained in collective sports (twice per week)	RCT, DB CG (*n* = 6)	Drop jumps (5 × 20, height: 60 cm)	12 days of supplementation (7 days before and 4 days after exercise). 2 × 10 g every day + 20 g before and after the exercise Amount of BCAA: 280 g (12 days) LEU/ISO/VAL (2:1:1) Placebo: aspartame based artificial sweeteners.	CG > SG at D1 and D2	CG > SG for CK (group effect considering the time range from D0 to D4)	SG > CG (group effect considering the time range from D0 to D4) for the MVC
Jackman et al. (2010) [36]	24 healthy males age: n/a training status: n/a	SB CG (*n* = 12) Dietary control	Eccentric exercise (12 × 10 knee extensions, 120% of 1 RM)	3 days of supplementation (1 supp before, 3 supp after exercise and 4 supp every day for 2 days) Amount of BCAA: 87.6g (3 days) LEU/ISO/VAL (2.1:1.2:1) Placebo: Artificially sweetened and flavored water	CG > SG with knee flexed at D2 and D3	CG = SG for CK and myoglobin	CG = SG
Kephart et al. (2016) [37]	30 healthy males age: 22 ± 1 years training status: resistance-trained	RCT CG (*n* = 15) Dietary control	3 back squat exercises on three consecutive days (10 × 5 at 80% of 1 RM)	4 days of supplementation (1 supp after the exercise on the first 3 days and 1 supp on day 4) Amount of BCAA: 24g (4 days) LEU/ISO/VAL (3:1:2) and CHO Placebo: CHO	CG = SG	CG = SG for myoglobin	CG = SG
Matsumoto et al. (2007) [38]	12 healthy subjects (males: *n* = 6 and female: *n* = 6) age: 20 ± 1 years training status: trained in long distance running	RCT DB Cross-over (washout: 3 weeks) Dietary control	7 sessions on 3 days of long distance runs	3 days of supplementation (20g/day) Amount of BCAA: 60g (3 days) LEU/ISO/VAL (2:1:1) Placebo: n/a	CG > SG at D1	CG > SG at D1 for CK, LDH and myoglobin	-
Ra et al. (2013) [39]	18 healthy male subjects age: 23 ± 1 years training status: n/a	RCT, DB CG (*n* = 9)	Eccentric exercise (6 × 5 elbow flexions, 90% of MVC)	18 days of supplementation (14 days before and 4 days after exercise). 3 × 3.2 g every day Amount of BCAA: 172.8g (18 days) LEU/ISO/VAL (2:1:1) Placebo: starch	CG = SG	CG = SG for CK and LDH	-
Shimomura et al. (2010) [40]	12 healthy female subjects age: 22 ± 2 years training status: untrained	Cross-over (washout: 11 weeks)	Resistance exercise (7 × 20 squat with body weight)	Single day supplementation (1 supp before the exercise) Amount of BCAA: 5.5g (1 day) LEU/ISO/VAL (2.3:1:1.2) Placebo: dextrin	CG > SG at D2 and D3	CG = SG for CK and myoglobin	SG > CG at D3 for MVC
Waldron et al. (2017) [41]	16 healthy subjects (males: *n* = 14 and female: *n* = 2) age: 22 ± 2 years training status: trained in resistance exercise	RCT CG (*n* = 8) Dietary control	Strength exercise (10 × 6 back squats at 70% of 1 RM)	3 days of supplementation (1 supp before, 1 supp after exercise and 2 supp every day for 2 days) Amount of BCAA: 48g (3 days) LEU/ISO/VAL (2:1:1) and dextrose Placebo: dextrose	CG = SG	SG > CG at D1 and D2 for CK	CG = SG for MVC and CMJ

LDH: lactate dehydrogenase; CK: creatine kinase; V̇O_2max_: maximal oxygen consumption; CG: control group; SG: supplemented group; RM: maximal repetition; H: hour (e.g., H4: four hours after the end of the damaging exercise); D: day (e.g., D4: four days after the damaging exercise); supp: supplementation; LEU: leucine; ISO: isoleucine; VAL: valine; CHO: carbohydrates; RCT: randomized clinical trial; DB: double blind; SB: single blind; MVC: maximal voluntary contraction force; CMJ: counter movement jump; SSPT: seated shot-put throw; n/a: not available.

**Table 3:** Quality assessment of included studies.

References	Validity Rating										Overall Rating
	1	2	3	4	5	6	7	8	9	10	
Coombes & McNaughton (2000) [31]	Y	Y	Y	N	N	Y	Y	Y	Y	N	ø
Fouré et al. (2016) [32]	Y	Y	Y	Y	Y	Y	Y	Y	Y	Y	+
Gee & Deniel (2016) [33]	Y	Y	N	N	N	N	Y	Y	Y	Y	ø
Greer et al. (2007) [34]	Y	Y	N	N	N	N	Y	N	Y	N	–
Howatson et al. (2012) [35]	Y	Y	Y	Y	Y	Y	Y	N	Y	Y	+
Jackman et al. (2010) [36]	Y	Y	Y	N	N	Y	Y	Y	Y	Y	+
Kephart et al. (2016) [37]	Y	Y	Y	N	N	Y	Y	Y	Y	Y	+
Matsumoto et al. (2007) [38]	Y	N	N	N	Y	Y	Y	Y	Y	N	ø
Ra et al. (2013) [39]	Y	Y	Y	N	Y	Y	Y	Y	Y	Y	+
Shimomura et al. (2010) [40]	Y	Y	N	N	N	N	Y	N	Y	N	–
Waldron et al. (2017) [41]	Y	Y	Y	N	Y	Y	Y	N	Y	Y	+
Total	11	10	6	2	5	8	11	7	11	7	

Validity items: 1 research question stated; 2 subject selection free from bias; 3 comparable study groups; 4 method for withdrawals described; 5 blinding used; 6 interventions described; 7 outcomes stated, measurements valid and reliable; 8 appropriate statistical analysis; 9 appropriate conclusions, limitations described; 10 funding and sponsorship free from bias. Validity items 2, 3, 6, 7 must be satisfied for a positive quality rating. Y: yes, N: no, +: positive, ø: neutral, –: negative.

**Table 4:** Rating of supplementation strategy and extent of muscle damage.

References	Extent of Muscle Damage	Supplementation Strategy		
		Duration	Frequency	Amount
Fouré et al. (2016) [32]	High	Moderate	Low	Low
Ra et al. (2013) [39]	High	Long	High	Low
Jackman et al. (2010) [36]	Moderate	Short	High	High
Coombes & McNaughton (2000) [31]	Moderate	Long	High	High
Howatson et al. (2012) [35]	Moderate	Long	High	High
Greer et al. (2007) [34]	Low	Short	Low	Low
Shimomura et al. (2010) [40]	Low	Short	Low	Low
Gee & Deniel (2016) [33]	Low	Short	Low	High
Matsumoto et al. (2007) [38]	Low	Short	High	High
Waldron et al. (2017) [41]	Low	Short	High	High
Kephart et al. (2016) [37]	Low	Moderate	Low	Low

**Table 5:** Muscle damage exercise outcomes of included studies.

References	Effects in the Control Group	Positive Effect of Supplementation
Muscle performance		
Fouré et al. (2016) [32]	Significant decrease in MVC from POST to D4	-
Gee & Deniel (2016) [33]	Significant decrease in CMJ and SSPT performances at D1	Yes
Greer et al. (2007) [34]	Significant decrease in torque (leg flexion and extension) from POST to D2	Yes
Howatson et al. (2012) [35]	Significant decrease in MVC from D1 to D3	Yes
Jackman et al. (2010) [36]	Significant decrease in maximal force from H1 to D3	-
Kephart et al. (2016) [37]	Significant decrease in isokinetic peak torque	-
Shimomura et al. (2010) [40]	Significant decrease in MVC at D3	Yes
Waldron et al. (2017) [41]	Decrease in MVC and CMJ performance from POST to D1	-
Blood analyses		
Coombes & McNaughton (2000) [31]	Significant increase in CK and LDH (from POST to D5)	Yes
Fouré et al. (2016) [32]	Significant increase in plasma CK activity at D3 and D4	-
Greer et al. (2007) [34]	Significant increase in CK (from H4 to D2) and LDH (at H4)	Yes
Howatson et al. (2012) [35]	Significant increase in CK from D1 to D3	Yes
Jackman et al. (2010) [36]	Significant increase in CK (from H8 to D3) and myoglobin (at H1, H8 and D3)	-
Kephart et al. (2016) [37]	Significant increase in myoglobin	-
Matsumoto et al. (2007) [38]	Significant increase in CK and LDH at POST	Yes
Ra et al. (2013) [39]	Significant increase in CK and LDH at D3 and D4	-
Shimomura et al. (2010) [40]	No significant change in CK and LDH on the three days post-exercise	-
Waldron et al. (2017) [41]	No change in CK on the two days post-exercise	-

LDH: lactate dehydrogenase, CK: creatine kinase, POST: immediately after the damaging exercise, H: hour (e.g., H4: four hours after the end of the damaging exercise), D: day (e.g., D4: four days after the damaging exercise), MVC: maximal voluntary contraction force, CMJ: counter movement jump, SSPT: seated shot-put throw.

## References

[1] Froiland K., Koszewski W., Hingst J., Kopecky L. (2004). Nutritional supplement use among college athletes and their sources of information. Int. J. Sport Nutr. Exerc. Metab..

[2] Huang S.H., Johnson K., Pipe A.L. (2006). The use of dietary supplements and medications by Canadian athletes at the Atlanta and Sydney olympic games. Clin. J. Sport Med..

[3] Tsitsimpikou C., Tsiokanos A., Tsarouhas K., Schamasch P., Fitch K.D., Valasiadis D., Jamurtas A. (2009). Medication use by athletes at the athens 2004 summer olympic games. Clin. J. Sport. Med..

[4] Goston J.L., Correia M.I. (2010). Intake of nutritional supplements among people exercising in gyms and influencing factors. Nutrition.

[5] Harper A.E., Miller R.H., Block K.P. (1984). Branched-chain amino acid metabolism. Annu. Rev. Nutr..

[6] Rennie M.J., Tipton K.D. (2000). Protein and amino acid metabolism during and after exercise and the effects of nutrition. Annu. Rev. Nutr..

[7] Valerio A., D’Antona G., Nisoli E. (2011). Branched-chain amino acids, mitochondrial biogenesis, and healthspan: An evolutionary perspective. Aging.

[8] Shimomura Y., Kobayashi H., Mawatari K., Akita K., Inaguma A., Watanabe S., Bajotto G., Sato J. (2009). Effects of squat exercise and branched-chain amino acid supplementation on plasma free amino acid concentrations in young women. J. Nutr. Sci. Vitaminol. (Tokyo).

[9] Matthews D.E. (2005). Observations of branched-chain amino acid administration in humans. J. Nutr..

[10] Mattick J.S., Kamisoglu K., Ierapetritou M.G., Androulakis I.P., Berthiaume F. (2013). Branched-chain amino acid supplementation: Impact on signaling and relevance to critical illness. Wiley Interdiscip. Rev. Syst. Biol. Med.

[11] Blomstrand E., Eliasson J., Karlsson H.K., Kohnke R. (2006). Branched-chain amino acids activate key enzymes in protein synthesis after physical exercise. J. Nutr..

[12] Kimball S.R., Jefferson L.S. (2006). Signaling pathways and molecular mechanisms through which branched-chain amino acids mediate translational control of protein synthesis. J. Nutr..

[13] Norton L.E., Layman D.K. (2006). Leucine regulates translation initiation of protein synthesis in skeletal muscle after exercise. J. Nutr..

[14] Dreyer H.C., Drummond M.J., Pennings B., Fujita S., Glynn E.L., Chinkes D.L., Dhanani S., Volpi E., Rasmussen B.B. (2008). Leucine-enriched essential amino acid and carbohydrate ingestion following resistance exercise enhances mtor signaling and protein synthesis in human muscle. Am. J. Physiol. Endocrinol. Metab..

[15] Wang X., Proud C.G. (2006). The mtor pathway in the control of protein synthesis. Physiology (Bethesda).

[16] D’Antona G., Ragni M., Cardile A., Tedesco L., Dossena M., Bruttini F., Caliaro F., Corsetti G., Bottinelli R., Carruba M.O. (2010). Branched-chain amino acid supplementation promotes survival and supports cardiac and skeletal muscle mitochondrial biogenesis in middle-aged mice. Cell Metab..

[17] Tatpati L.L., Irving B.A., Tom A., Bigelow M.L., Klaus K., Short K.R., Nair K.S. (2010). The effect of branched chain amino acids on skeletal muscle mitochondrial function in young and elderly adults. J. Clin. Endocrinol. Metab..

[18] Doi J., Shiraishi K., Haida M., Matsuzaki S. (2004). Abnormality of energy metabolism in the skeletal muscle of patients with liver cirrhosis and changes under administration of glucose and branched-chain amino acids. Tokai J. Exp. Clin. Med..

[19] Kutsuzawa T., Kurita D., Haida M. (2011). Acute effects of branched-chain amino acids on muscle pH during exercise. Adv. Exerc. Sports Physiol..

[20] Spriet L., Hargreaves M., Spriet L. (2006). Anaerobic metabolism during exercise. Exercise Metabolism.

[21] Clarkson P.M., Hubal M.J. (2002). Exercise-induced muscle damage in humans. Am. J. Phys. Med. Rehabil..

[22] Lieber R.L., Friden J. (1993). Muscle damage is not a function of muscle force but active muscle strain. J. Appl. Physiol..

[23] Armstrong R.B. (1984). Mechanisms of exercise-induced delayed onset muscular soreness: A brief review. Med. Sci. Sports Exerc..

[24] MacLean D.A., Graham T.E., Saltin B. (1994). Branched-chain amino acids augment ammonia metabolism while attenuating protein breakdown during exercise. Am. J. Physiol..

[25] Fouré A., Duhamel G., Wegrzyk J., Boudinet H., Mattei J.P., Le Troter A., Bendahan D., Gondin J. (2015). Heterogeneity of muscle damage induced by electrostimulation: A multimodal mri study. Med. Sci. Sports Exerc..

[26] Fouré A., Wegrzyk J., Le Fur Y., Mattei J.P., Boudinet H., Vilmen C., Bendahan D., Gondin J. (2015). Impaired mitochondrial function and reduced energy cost as a result of muscle damage. Med. Sci. Sports Exerc..

[27] Nair K.S., Schwartz R.G., Welle S. (1992). Leucine as a regulator of whole body and skeletal muscle protein metabolism in humans. Am. J. Physiol..

[28] Mackey A.L., Bojsen-Moller J., Qvortrup K., Langberg H., Suetta C., Kalliokoski K.K., Kjaer M., Magnusson S.P. (2008). Evidence of skeletal muscle damage following electrically stimulated isometric muscle contractions in humans. J. Appl. Physiol..

[29] Liberati A., Altman D.G., Tetzlaff J., Mulrow C., Gotzsche P.C., Ioannidis J.P., Clarke M., Devereaux P.J., Kleijnen J., Moher D. (2009). The prisma statement for reporting systematic reviews and meta-analyses of studies that evaluate health care interventions: Explanation and elaboration. PLoS Med..

[30] AND Evidence Analysis Manual: Steps in the Academiy Evidence Analysis Process. https://www.andeal.org/vault/2440/web/files/QCC_3.pdf.

[31] Coombes J.S., McNaughton L.R. (2000). Effects of branched-chain amino acid supplementation on serum creatine kinase and lactate dehydrogenase after prolonged exercise. J. Sports Med. Phys. Fitness.

[32] Fouré A., Nosaka K., Gastaldi M., Mattei J.P., Boudinet H., Guye M., Vilmen C., Le Fur Y., Bendahan D., Gondin J. (2016). Effects of branched-chain amino acids supplementation on both plasma amino acids concentration and muscle energetics changes resulting from muscle damage: A randomized placebo controlled trial. Clin. Nutr..

[33] Gee T.I., Deniel S. (2016). Branched-chain aminoacid supplementation attenuates a decrease in power-producing ability following acute strength training. J. Sports Med. Phys. Fitness.

[34] Greer B.K., Woodard J.L., White J.P., Arguello E.M., Haymes E.M. (2007). Branched-chain amino acid supplementation and indicators of muscle damage after endurance exercise. Int. J. Sport Nutr. Exerc. Metab..

[35] Howatson G., Hoad M., Goodall S., Tallent J., Bell P.G., French D.N. (2012). Exercise-induced muscle damage is reduced in resistance-trained males by branched chain amino acids: A randomized, double-blind, placebo controlled study. J. Int. Soc. Sports Nutr..

[36] Jackman S.R., Witard O.C., Jeukendrup A.E., Tipton K.D. (2010). Branched-chain amino acid ingestion can ameliorate soreness from eccentric exercise. Med. Sci. Sports Exerc..

[37] Kephart W.C., Mumford P.W., McCloskey A.E., Holland A.M., Shake J.J., Mobley C.B., Jagodinsky A.E., Weimar W.H., Oliver G.D., Young K.C. (2016). Post-exercise branched chain amino acid supplementation does not affect recovery markers following three consecutive high intensity resistance training bouts compared to carbohydrate supplementation. J. Int. Soc. Sports Nutr..

[38] Matsumoto K., Mizuno M., Mizuno T., Dilling-Hansen B., Lahoz A., Bertelsen V., Munster H., Jordening H., Hamada K., Doi T. (2007). Branched-chain amino acids and arginine supplementation attenuates skeletal muscle proteolysis induced by moderate exercise in young individuals. Int. J. Sports Med..

[39] Ra S.G., Miyazaki T., Ishikura K., Nagayama H., Komine S., Nakata Y., Maeda S., Matsuzaki Y., Ohmori H. (2013). Combined effect of branched-chain amino acids and taurine supplementation on delayed onset muscle soreness and muscle damage in high-intensity eccentric exercise. J. Int. Soc. Sports Nutr..

[40] Shimomura Y., Inaguma A., Watanabe S., Yamamoto Y., Muramatsu Y., Bajotto G., Sato J., Shimomura N., Kobayashi H., Mawatari K. (2010). Branched-chain amino acid supplementation before squat exercise and delayed-onset muscle soreness. Int. J. Sport Nutr. Exerc. Metab..

[41] Waldron M., Whelan K., Jeffries O., Burt D., Howe L., Patterson S.D. (2017). The effects of acute branched-chain amino acid supplementation on recovery from a single bout of hypertrophy exercise in resistance-trained athletes. Appl. Physiol. Nutr. Metab..

[42] Pasiakos S.M., McLellan T.M., Lieberman H.R. (2015). The effects of protein supplements on muscle mass, strength, and aerobic and anaerobic power in healthy adults: A systematic review. Sports Med..

[43] Warren G.L., Lowe D.A., Armstrong R.B. (1999). Measurement tools used in the study of eccentric contraction-induced injury. Sports Med..

[44] Clarkson P.M., Ebbeling C. (1988). Investigation of serum creatine kinase variability after muscle-damaging exercise. Clin. Sci. (Lond.).

[45] Fouré A., Nosaka K., Wegrzyk J., Duhamel G., Le Troter A., Boudinet H., Mattei J.P., Vilmen C., Jubeau M., Bendahan D. (2014). Time course of central and peripheral alterations after isometric neuromuscular electrical stimulation-induced muscle damage. PLoS ONE.

[46] Revill S.I., Robinson J.O., Rosen M., Hogg M.I. (1976). The reliability of a linear analogue for evaluating pain. Anaesthesia.

[47] Ohnhaus E.E., Adler R. (1975). Methodological problems in the measurement of pain: A comparison between the verbal rating scale and the visual analogue scale. Pain.

[48] Nosaka K., Newton M., Sacco P. (2002). Delayed-onset muscle soreness does not reflect the magnitude of eccentric exercise-induced muscle damage. Scand. J. Med. Sci. Sports.

[49] Zory R., Boerio D., Jubeau M., Maffiuletti N.A. (2005). Central and peripheral fatigue of the knee extensor muscles induced by electromyostimulation. Int. J. Sports Med..

[50] Martin V., Millet G.Y., Lattier G., Perrod L. (2004). Effects of recovery modes after knee extensor muscles eccentric contractions. Med. Sci. Sports Exerc..

[51] Prasartwuth O., Taylor J.L., Gandevia S.C. (2005). Maximal force, voluntary activation and muscle soreness after eccentric damage to human elbow flexor muscles. J. Physiol..

[52] Behrens M., Mau-Moeller A., Bruhn S. (2012). Effect of exercise-induced muscle damage on neuromuscular function of the quadriceps muscle. Int. J. Sports Med..

[53] Fouré A., Le Troter A., Guye M., Mattei J.P., Bendahan D., Gondin J. (2015). Localization and quantification of intramuscular damage using statistical parametric mapping and skeletal muscle parcellation. Sci. Rep..

[54] Lacourpaille L., Nordez A., Hug F., Couturier A., Dibie C., Guilhem G. (2014). Time-course effect of exercise-induced muscle damage on localized muscle mechanical properties assessed using elastography. Acta Physiol. (Oxf.).

[55] Guilhem G., Hug F., Couturier A., Regnault S., Bournat L., Filliard J.R., Dorel S. (2013). Effects of air-pulsed cryotherapy on neuromuscular recovery subsequent to exercise-induced muscle damage. Am. J. Sports Med..

[56] Nosaka K., Sakamoto K. (2001). Effect of elbow joint angle on the magnitude of muscle damage to the elbow flexors. Med. Sci. Sports Exerc..

[57] Davies R.C., Eston R.G., Fulford J., Rowlands A.V., Jones A.M. (2011). Muscle damage alters the metabolic response to dynamic exercise in humans: A 31p-mrs study. J. Appl. Physiol..

[58] Sorichter S., Koller A., Haid C., Wicke K., Judmaier W., Werner P., Raas E. (1995). Light concentric exercise and heavy eccentric muscle loading: Effects on ck, mri and markers of inflammation. Int. J. Sports Med..

[59] Aboodarda S.J., George J., Mokhtar A.H., Thompson M. (2011). Muscle strength and damage following two modes of variable resistance training. J. Sports Sci. Med..

[60] Macaluso F., Isaacs A.W., Myburgh K.H. (2012). Preferential type ii muscle fiber damage from plyometric exercise. J. Athl. Train..

[61] Areces F., Salinero J.J., Abian-Vicen J., Gonzalez-Millan C., Gallo-Salazar C., Ruiz-Vicente D., Lara B., Del Coso J. (2014). A 7-day oral supplementation with branched-chain amino acids was ineffective to prevent muscle damage during a marathon. Amino Acids.

[62] Pasiakos S.M., Lieberman H.R., McLellan T.M. (2014). Effects of protein supplements on muscle damage, soreness and recovery of muscle function and physical performance: A systematic review. Sports Med..

[63] Nosaka K., Sacco P., Mawatari K. (2006). Effects of amino acid supplementation on muscle soreness and damage. Int. J. Sport Nutr. Exerc. Metab..

